# Supplementary material for: The genome sequence of a tachinid fly, Dexiosoma caninum (Fabricius, 1781)
Source: Wellcome Open Res. 2024 Oct 15;9:595. [Version 1] doi: 10.12688/wellcomeopenres.23193.1 (PMC12203045; doi:10.12688/wellcomeopenres.23193.1)
Supplement: Peer review history

# Reviewer report: Reviewer response for version 1

**DOI:** 10.21956/wellcomeopenres.25543.r110709

**Published:** 2025-06-26

**Authors:** Marcus Stensmyr

**Affiliations:** 1 Lund University, Lund, Sweden

The submitted manuscript presents a high-quality genome assembly of a tachinid fly *Dexiosoma caninum*, sequenced as part of the Darwin Tree of Life project (DToL). The genome has been sequenced and assembled using the state-of-the-art pipeline employed by the DToL project. The genome is, however, not annotated, which makes this resource less useful. Ideally the authors should run the standard Ensembl pipeline. A genome assembly of a Tachinid fly is a welcome addition to the field of Diptera genomics.

Are sufficient details of methods and materials provided to allow replication by others?

Yes

Is the rationale for creating the dataset(s) clearly described?

Yes

Are the datasets clearly presented in a useable and accessible format?

Yes

Are the protocols appropriate and is the work technically sound?

Yes

Reviewer Expertise:

Diptera genomics

I confirm that I have read this submission and believe that I have an appropriate level of expertise to confirm that it is of an acceptable scientific standard.

# Reviewer report: Reviewer response for version 1

**DOI:** 10.21956/wellcomeopenres.25543.r108177

**Published:** 2024-11-22

**Authors:** Henrique Antoniolli

**Affiliations:** 1 Universidade Federal do Rio Grande do Sul (UFRGS), Porto Alegre, Brazil

The present study reports a highly contiguous genome assembly of *Dexiosoma caninum*. The background section describes the species and what is known about its biology, including the reasons for sequencing its genome. Methods and protocols are well described. The produced data are deposited in public repositories, which accession codes are clearly stated throughout the text. Finally, all statistics about the genome assembly achieved satisfactory results. In this sense, I recommend indexing this paper.

Are sufficient details of methods and materials provided to allow replication by others?

Yes

Is the rationale for creating the dataset(s) clearly described?

Yes

Are the datasets clearly presented in a useable and accessible format?

Yes

Are the protocols appropriate and is the work technically sound?

Yes

Reviewer Expertise:

Genomics, evolution

I confirm that I have read this submission and believe that I have an appropriate level of expertise to confirm that it is of an acceptable scientific standard.
